# Biology’s transformation: from observation through experiment to computation

**DOI:** 10.1093/bioadv/vbae069

**Published:** 2024-05-22

**Authors:** Christos A Ouzounis

**Affiliations:** Biological Computation & Computational Biology Group, Artificial Intelligence & Information Analysis Laboratory, School of Informatics, Faculty of Sciences, Aristotle University of Thessalonica, Thessalonica GR-54124, Greece

## Abstract

**Summary:**

We explore the nuanced temporal and epistemological distinctions among natural sciences, particularly the contrasting treatment of time and the interplay between theory and experimentation. Physics, an exemplar of mature science, relies on theoretical models for predictability and simulations. In contrast, biology, traditionally experimental, is witnessing a computational surge, with data analytics and simulations reshaping its research paradigms. Despite these strides, a unified theoretical framework in biology remains elusive. We propose that contemporary global challenges might usher in a renewed emphasis, presenting an opportunity for the establishment of a novel theoretical underpinning for the life sciences.

**Availability and implementation:**

https://github.com/ouzounis/CLS-emerges Data in Json format, Images in PNG format.

The natural sciences exhibit notable distinctions, particularly regarding the role of time in a historical context. Mathematics, physics, and chemistry possess the ability to “reverse” time, as much of their subject matter is theoretically, or even practically, reversible. On the other hand, sciences like biology, geology, and astronomy inherently incorporate historicity, where past events significantly influence our present observations and time equations (Montévil 2022). Another crucial epistemological distinction shaping scientific research is whether a discipline leans toward experimentation or theory. The latter finds its prime examples in fundamental mathematics and physics. Experimental sciences encompass a broad range from chemistry and biology to geology and astronomy, with the latter two sometimes categorized as observational sciences due to the constraints on conducting certain experiments at scale (Daston 2008). The progression of a scientific discipline is often delineated by its level of “maturity” (Bunge 1968), starting with observation, advancing to experimentation, and ultimately culminating in computation, with theory and applications. Therefore, physics has traditionally been seen as the pinnacle of the natural sciences, and perhaps rightly so. Meanwhile, biology has primarily been classified as an experimental science with elements of historicity, thus leaning toward observation or experiment and not being perceived as a more mature, theoretical science. Recent developments in the last few decades have been causing a shift in this perspective.

A “mature” science, like physics, is defined by the development of theoretical frameworks that enable a high degree of predictability within the systems being studied. These theories encompass everything from simple “laws,” such as those governing gravitational acceleration, to intricate “simulations” exploring emergent properties (Varley and Hoel 2022). The primary function of a theory is to establish potential space–time trajectories, reducing or eliminating altogether the necessity of conducting certain experiments. Should an experimenter choose to carry out an experiment adhering to the framework’s strict reproducibility criteria, the theory should accurately foresee the experimental outcome. If these predictions do not align with the experimental findings, scientists will search for modifications to the existing framework or even propose an entirely new theory (Kuhn 1962). In today’s complex scientific landscape, computational methods often take on a key role either in putting theory into practice or serving as substitutes for theoretical approaches. These methods resemble virtual simulations of experimental scenarios, supported by a mix of equations, statistics, and empirical data or observations (Frické 2015). The ability to make generalized predictions is crucial, as the maturity of a theory is evident in its capacity to predict the behavior of intricate phenomena, systems, or processes. This predictive nature serves as a testament to the effectiveness of the more mature, theory-based sciences.

Biology, much like physics in its earlier stages, originated from observations and experiments. While there have been philosophical debates regarding whether biology strictly adheres to the laws of physics or further possesses its own, unique laws (Rosen 1987, Dhar and Giuliani 2010), the foundation of modern biology rests on two principal pillars: the study of structure as genetics (life’s mathematics) and the investigation of function as biochemistry (life’s chemistry). A pivotal achievement in early 20th century biology was the development of the Modern Synthesis, which integrated the theory of evolution by natural selection with the theory of inheritance, as epitomized by the iconic works of Darwin and Mendel, respectively (Mayr 1993). A generation or two ago, the manipulation of biological systems via genetic engineering marked the rise of molecular biology (Stent 1968). Subsequently, biological science underwent a shift with more systematic studies and advancements in technology that ushered in the Genome Era (Botstein 2011). Now, the question arises: What is the present-day epistemological status of biology? Will it predominantly remain an experimental science, or is it rapidly moving toward “maturity,” supported by computation?

Over the course of four decades, a central question has emerged concerning the transformation of biology into a science more akin to physics than to natural history, and the role that bioinformatics has played in this process (Ouzounis 2012). With strong affirmation, which is widely held by many, biology has unquestionably attained a notable level of sophistication, where extensive data analytics enable us to make solid predictions for various outcomes without solely relying on experiments (Markowetz 2017). Research in the life sciences now includes more theoretical work than outsiders might recognize, involving substantial computational demands, algorithmic depth, massive volumes of data, and the integration of robotic automation into large-scale experiments. This evolving landscape suggests that biology is entering a digital phase where experiments are increasingly guided by computation and modeling. Thanks to the growing predictive power of simulations, they encompass a multitude of potential experiments that need not be physically conducted, with the incorporation of machine learning.

Thus, contemporary life science research increasingly relies on automated procedures and high data volumes, driven by the rapid development of sequencing technologies, and slowly moving away from traditional natural history-based observations or hypothesis-based, classical experiments. Relevant observations within the life sciences span diverse scales, ranging from the molecular level with precision down to individual atoms, through microscales utilizing advanced imaging technologies, to macroscales facilitated by planetary-level sensors, as well as collaborative efforts like observatories, scientific networks, and crowdsourcing initiatives (Nathan *et al.* 2022). These efforts are often supported or inspired by other disciplines, such as the earth sciences.

While biology is not exclusively a computational science, it is undoubtedly a broad discipline that heavily relies on computation by any standard (Fig. 1). Computational methods are extensively employed to control experiments across various levels, and the intricacies of life continuously stimulate innovative, “bio-inspired” design approaches for both hardware and software engineering. However, what remains absent is a comprehensive “grand” theory that can encompass the recurrent observations and predictable behaviors observed in biological systems (Krakauer *et al.* 2011). Such a theory would typically rely on CPU- and data-intensive systems in the short term, as well as innovative conceptual frameworks in the long term.

**Figure 1.**
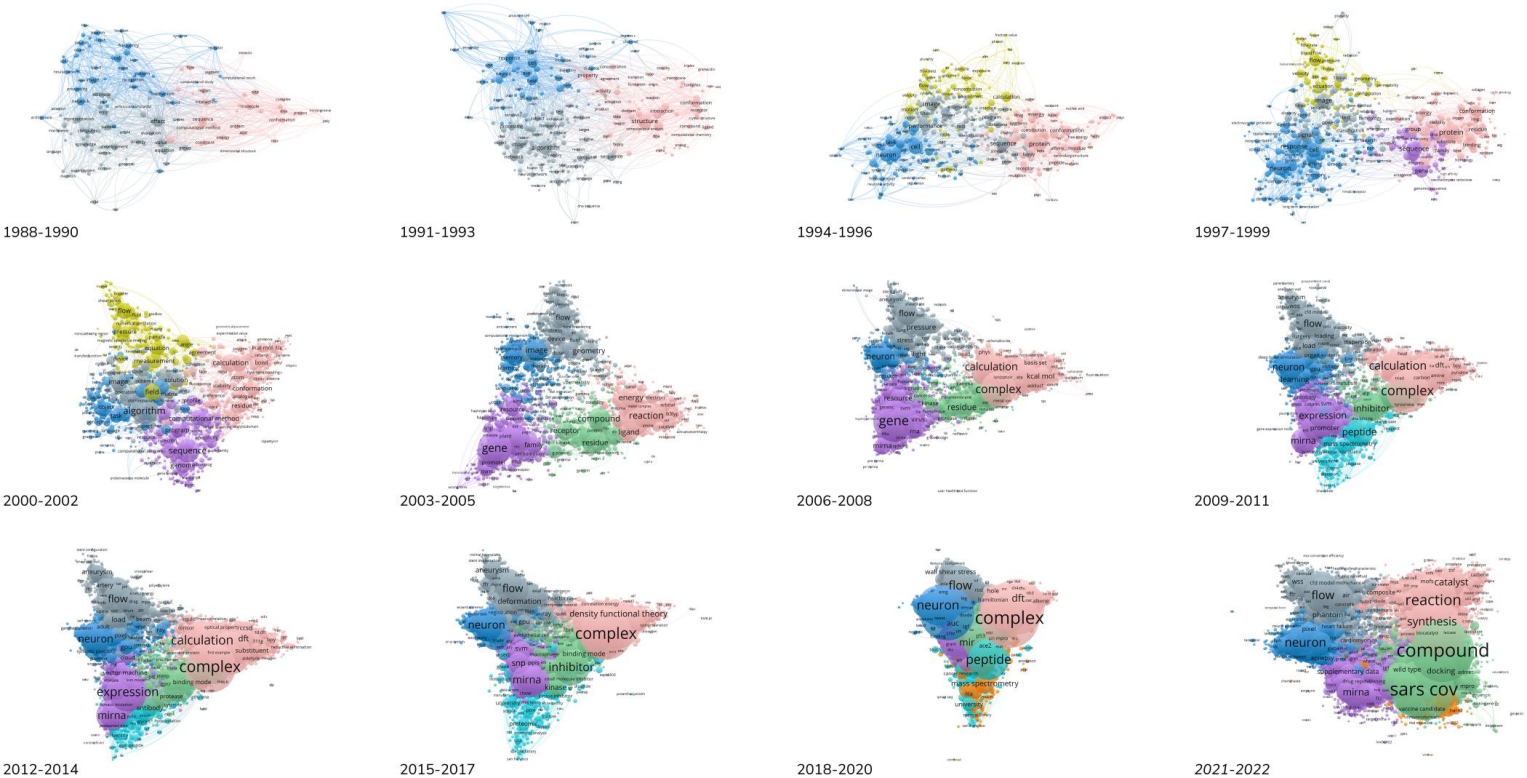
Evolution of terms in the literature associated with the term “computational” and over 3-year periods from 1988 to 2022 (last period lasts two years, 2023 is excluded). Color code: pink—“biocomputing”, bluegray—toolkits to robots, seafoam—neurocomputing, olive—medical devices, purple—genome informatics, seagreen—pharmacogenomics, skyblue—proteomics, orange—immunogenomics (see GitHub README for details). Generated with VOSviewer (Van Eck and Waltman 2010). Data type: map on text data, data source: Europe PMC, search query: title + abstract, counting: full, minimum number of occurrences: 10, relevance score percentage: 60. See also Supplementary Figures S1 and S2. Results are reproducible and not affected significantly with change of parameters.

Furthermore, there is an opportunity to cultivate a similar social and political momentum as when physics dominated in the past. Recent global trends such as pandemics, conflicts, the climate crisis, widespread environmental destruction, and health challenges posed by an ageing population or wealth imbalance could offer the socio-political context needed for a new era of increased funding, akin to what physics has received historically. This renewed drive could contribute to the development of a comprehensive framework for the biological sciences while simultaneously aiding in the management and restoration of natural ecosystems, with a wide array of applications aimed at promoting human and ecosystem well-being in the 21st century.

## Supplementary Material

vbae069_Supplementary_Data

## Data Availability

The data underlying this article is available on GitHub at https://github.com/ouzounis/CLS-emerges: data in JSON format and images in PNG format.

## References

[vbae069-B1] Botstein D. Fruits of genome sequences for biology. Science 2011;331:1025. 10.1126/science.1204038 21350164

[vbae069-B2] Bunge M. The maturation of science. In: Lakatos I, Musgrave A (eds), Studies in Logic and the Foundations of Mathematics, Vol. 49. Elsevier, 1968, 120–47.

[vbae069-B3] Daston L. On scientific observation. ISIS 2008;99:97–110.

[vbae069-B4] Dhar PK , Giuliani A. Laws of biology: why so few? Syst Synth Biol 2010;4:7–13. 20186254 10.1007/s11693-009-9049-0 PMC2816229

[vbae069-B5] Frické M. Big data and its epistemology. J Assoc Inf Sci Technol 2015;66:651–61.

[vbae069-B6] Krakauer DC , Collins JP, Erwin D et al The challenges and scope of theoretical biology. J Theor Biol 2011;276:269–76. 21315730 10.1016/j.jtbi.2011.01.051

[vbae069-B7] Kuhn TS. The Structure of Scientific Revolutions, 3rd edn. Chicago, IL: University of Chicago Press, 1962.

[vbae069-B8] Markowetz F. All biology is computational biology. PLoS Biol 2017;15:e2002050. 28278152 10.1371/journal.pbio.2002050 PMC5344307

[vbae069-B9] Mayr E. What was the evolutionary synthesis? Trends Ecol Evol 1993;8:31–4. 21236096 10.1016/0169-5347(93)90128-C

[vbae069-B10] Montévil M. Historicity at the heart of biology. Theory Biosci 2022;141:165–73. 32613275 10.1007/s12064-020-00320-8

[vbae069-B11] Nathan R , Monk CT, Arlinghaus R et al Big-data approaches lead to an increased understanding of the ecology of animal movement. Science 2022;375:eabg1780. 35175823 10.1126/science.abg1780

[vbae069-B12] Ouzounis CA. Rise and demise of bioinformatics? Promise and progress. PLoS Comput Biol 2012;8:e1002487. 22570600 10.1371/journal.pcbi.1002487 PMC3343106

[vbae069-B13] Rosen R. Some epistemological issues in physics and biology. In: Hiley B, Peat FD (eds), Quantum Implications. New York, NY: Routledge, 1987, 314–27.

[vbae069-B14] Stent GS. That was the molecular biology that was. Science 1968;160:390–5. 4868510 10.1126/science.160.3826.390

[vbae069-B15] Van Eck NJ , Waltman L. VOSviewer, a computer program for bibliometric mapping. Scientometrics 2010;84:523–38. 20585380 10.1007/s11192-009-0146-3 PMC2883932

[vbae069-B16] Varley TF , Hoel E. Emergence as the conversion of information: a unifying theory. Philos Trans A Math Phys Eng Sci 2022;380:20210150. 35599561 10.1098/rsta.2021.0150 PMC9131462

