# Supplementary figures and images for: Biology’s transformation: from observation through experiment to computation
Source: Bioinform Adv. 2024 May 22;4(1):vbae069. doi: 10.1093/bioadv/vbae069 (PMC11127110; doi:10.1093/bioadv/vbae069)

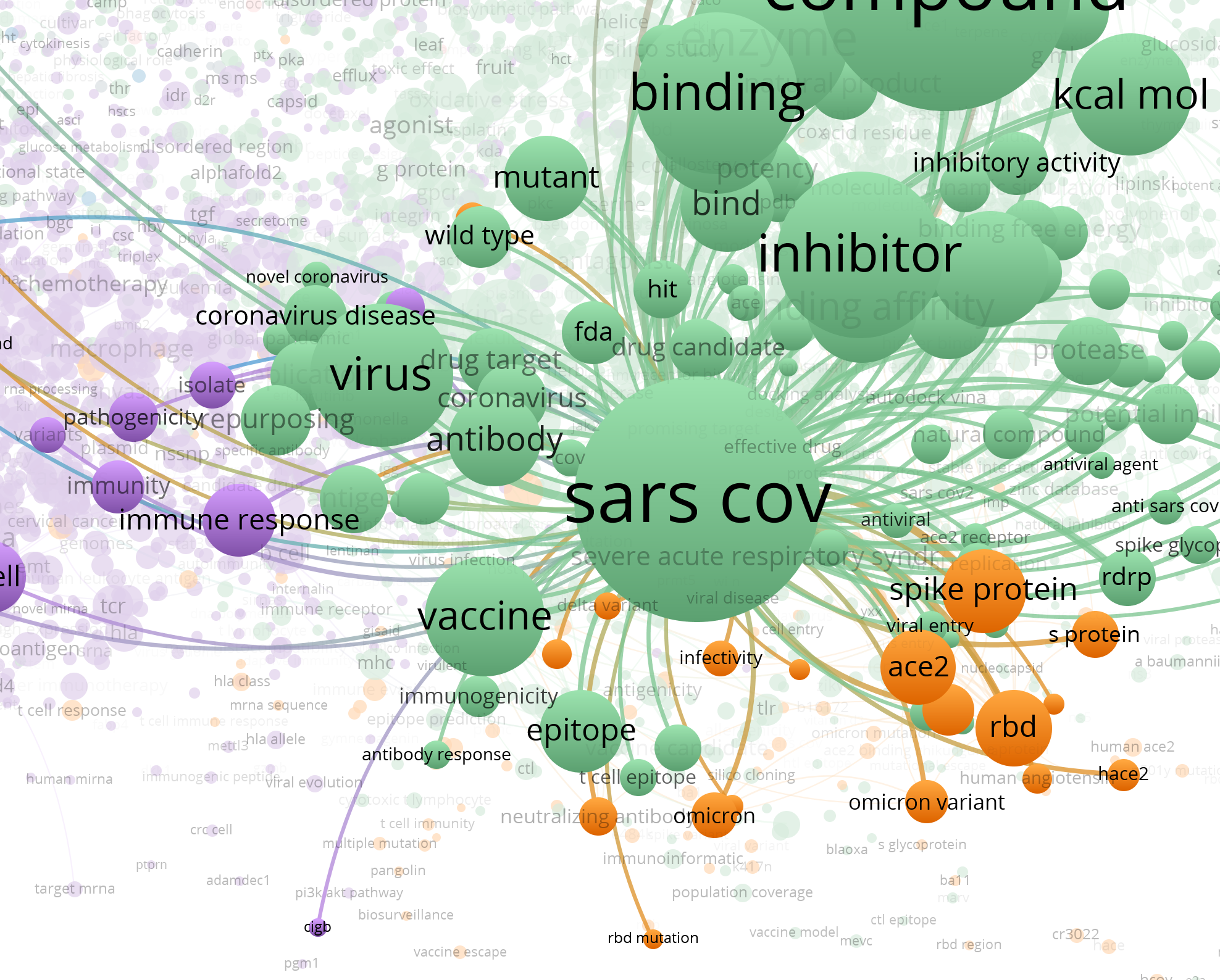

Supplement: vbae069_Supplementary_Data [file vbae069_supplementary_data.zip › 2022.coronavirus.png]

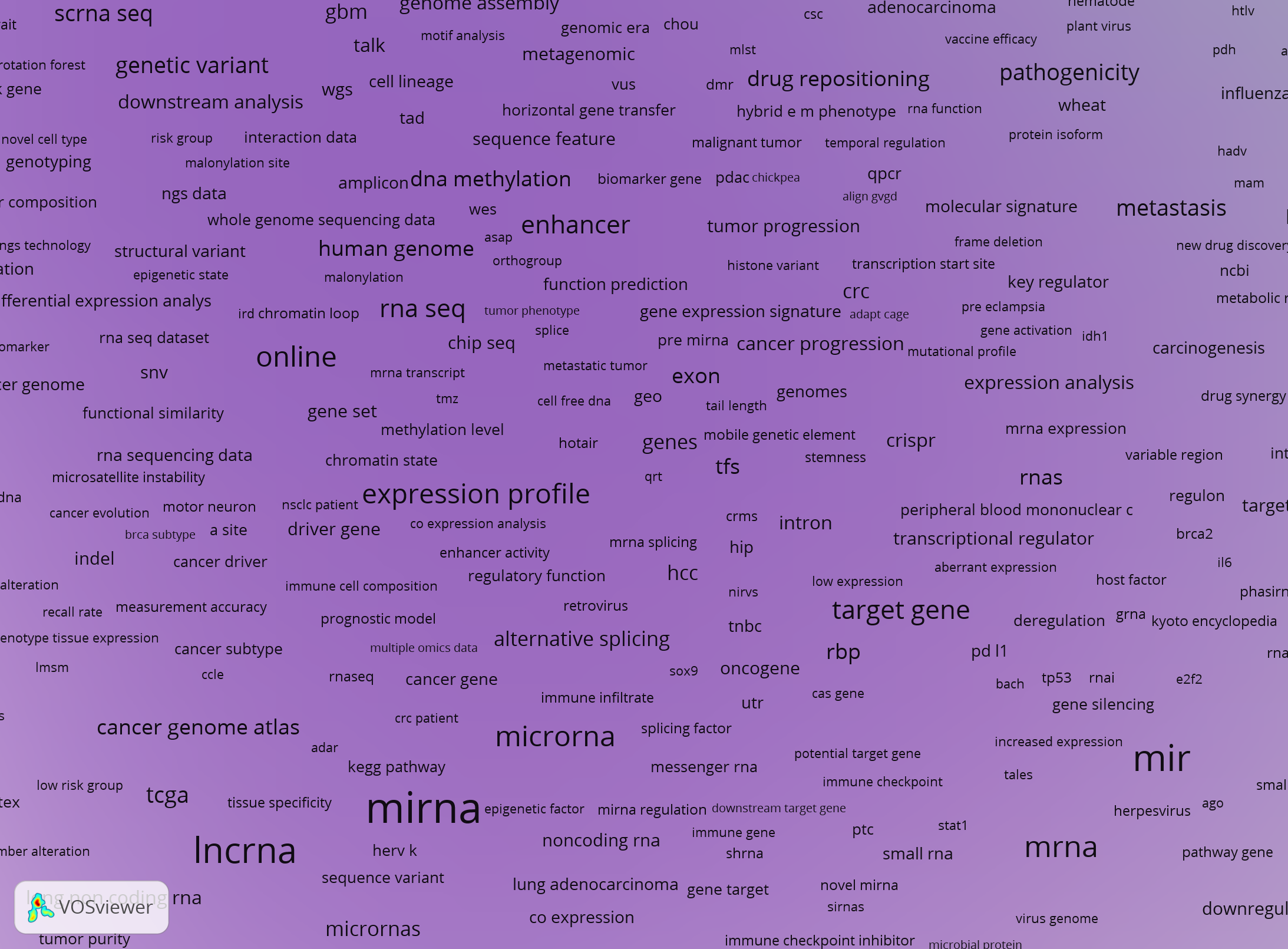

Supplement: vbae069_Supplementary_Data [file vbae069_supplementary_data.zip › 2020.genomics.png]
